# Evaluating the Cost-Effective Use of Follow-Up Colonoscopy Based on Screening Findings and Age

**DOI:** 10.1155/2019/2476565

**Published:** 2019-02-19

**Authors:** Grace N. Joseph, Farid Heidarnejad, Eric A. Sherer

**Affiliations:** Louisiana Tech University, Ruston, LA, USA

## Abstract

**Introduction:**

Colorectal cancer (CRC), if not detected early, can be costly and detrimental to one's health. Colonoscopy can identify CRC early as well as prevent the disease. The benefit of screening colonoscopy has been established, but the optimal frequency of follow-up colonoscopy is unknown and may vary based on findings from colonoscopy screening and patient age.

**Methods:**

A partially observed Markov process (POMP) was used to simulate the effects of follow-up colonoscopy on the development of CRC. The POMP uses adenoma and CRC growth models to calculate the probability of a patient having colorectal adenomas and CRC. Then, based on mortality, quality of life, and the costs associated with diagnosis, treatment, and surveillance of colorectal cancer, the overall costs and increase in quality-adjusted life years (QALYs) are calculated for follow-up colonoscopy scenarios.

**Results:**

At the $100,000/QALY gained threshold, only one follow-up colonoscopy is cost-effective only after screening at age 50 years. The optimal follow-up is 8.5 years, which gives 84.0 QALYs gained/10,000 persons. No follow-up colonoscopy was cost-effective at the $50,000 and $75,000/QALY gained thresholds. The intervals were insensitive to the findings at screening colonoscopy.

**Conclusion:**

Follow-up colonoscopy is cost-effective following screening at age 50 years but not if screening occurs later. Following screening at age 50 years, the optimal follow-up interval is close to the currently recommended 10 years for an average risk screening but does not vary by colonoscopy result.

## 1. Introduction

Colorectal cancer (CRC), if not detected early, can be costly and detrimental to one's health. CRC is the third most commonly diagnosed cancer and also the third leading cause of cancer-related deaths in both men and women in the United States [1] with about 90 percent of CRC cases developing in persons 50 years and older [2]. But CRC is both treatable and preventable when detected at an early stage [1]. Appropriate screening exams can find and remove precancerous adenomas in an effort to prevent future CRC. Several screening techniques have emerged over decades in aid of helping lower the disease [3]. Colonoscopy screening with the removal of adenomas is an effective strategy for reducing CRC incidence and mortality [4].

The Micro-Simulation Screening Analysis Colonoscopy (MISCAN-Colon) and Simulation Model of CRC (SimCRC) models were two simulation models that compared strategies for screening that vary by age [5, 6]. Based on such simulation models and clinical evidence, the US Preventive Task Force (USPTF) guidelines recommend that patients at average risk of colorectal cancer be screened starting at age 50 and should end at age 75 years [6, 7]. The USPTF also recommends that a follow-up colonoscopy should be received after 10 years if no adenomas are found, 5–10 years if 1-2 small adenomas are found, and 3 years if >3 adenomas are found at screening colonoscopy [8]. While simulation studies have been done to evaluate the cost-effectiveness of CRC screening, the cost-effectiveness of follow-up colonoscopy has not been evaluated. This is an important issue because once a person undergoes screening colonoscopy, there is potential for a lifetime of follow-up colonoscopy and, due to the CRC prevention ability of colonoscopy, the CRC detection and prevention benefits of colonoscopy may decrease after an initial screening colonoscopy.

Because the incidence of CRC is age dependent, the effectiveness at reducing mortality due to CRC and the varying costs associated with the disease depends on the age at which the first colonoscopy is performed [9]. Although it has been found that colonoscopy screening is effective at reducing CRC incidence and mortality [10], the effectiveness comes at a significant additional cost [11]. Therefore, the objective of this study is to identify the balance between intercolonoscopy interval and costs to determine the cost-effective intercolonoscopy interval that maximizes the gain in quality-adjusted life years based on findings from screening colonoscopy and a patient's age.

## 2. Methods

**Figure 1:**
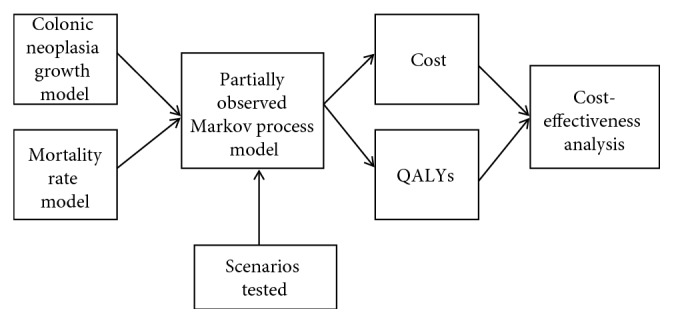
General overview of simulation process.

An overview of the simulation process is shown in Figure 1. A partially observed Markov process (POMP) was used to simulate the effects that intervening with different follow-up colonoscopy scenarios had on the development of CRC. The POMP uses adenoma and CRC natural history growth models to calculate the probability of a patient having colorectal adenomas; asymptomatic local, regional, or distant CRC; and symptomatic local, regional, or distant CRC as the patient ages. Some of these adenomas and CRCs are then detected and removed during colonoscopy with the detection rate depending on the size of the neoplasia. The natural history growth models are coupled to a mortality rate model that calculates the probability of dying due to natural causes or CRC-related mortality. Then, based on mortality, quality of life, and the costs associated with diagnosis, treatment, and surveillance of colorectal cancer, the overall costs and benefits in terms of quality-adjusted life years (QALYs) are calculated for each follow-up colonoscopy scenario to determine its effects on the cost-effectiveness and QALYs gained.

### 2.1. Partially Observed Markov Process

#### 2.1.1. Colonic Neoplasia Growth Model

**Figure 2:**
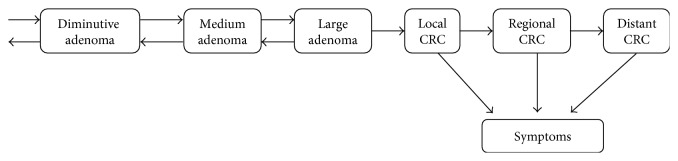
Adenoma growth model: adenoma growth [12], CRC growth [13], and development of symptoms [14].

The colonic neoplasia natural history progression model combined transition rates from two studies each focusing on different phases of colonic neoplasia development: adenoma growth and CRC growth (Figure 2).

To model adenoma growth, Sherer et al. [12] used serial colonoscopy results to identify the transition rates for the series of transitions from diminutive adenoma (<5 mm) to medium adenoma (6–9 mm) to large/advanced adenoma (>10 mm) to CRC (Figure 2). It was assumed that multiple colorectal neoplasia can exist and grow/regress and that both the growth of each neoplasia and the appearance of new adenomas are independent of the other neoplasia. They tested whether each rate was age dependent and found that the rate of appearance of new adenomas varied with patients' age but the transitions to more advanced neoplasia were age independent. The rates obtained from Sherer et al. were integrated over one-month intervals to get the monthly transition probabilities of individual transitions, and the monthly transition probabilities were combined to get the yearly transition probabilities (Table 1).

To model CRC growth, the MISCAN-Colon model used SEER CRC prevalence data to identify the transition rates between CRC stages [13]. Each combination of adenomas and CRC defines a possible state of the colon. For example, the *i* ^th^ state, *x*_*i*_=[*N*_*d*,*i*_, *N*_*m*,*i*_, …], is given by *N*_*d*,*i*_ number of diminutive adenomas, *N*_*m*,*i*_ number of medium adenomas, etc. The probability that a patient is in the *i* ^th^ state at age *t* is *P*_*i*_(*t*). After a time period, Δ*t*, a patient in the *i* ^th^ state can transition to any other possible state *j* (including remaining in the *i* ^th^ state) with a probability *K*_*i*,*j*_(*t*). Assuming no interventions, the dynamics of colonic state probability vector is the Markov process:

(1) $$\begin{array}{c} P\left(t+\Delta t\right)=\mathrm{KP}\left(t\right), \end{array}$$

where, initially at age 0 years, there are no neoplasia. Then, as a person ages, the risk of CRC increases as adenomas develop, grow, and transition to CRC.

As patients age, symptoms can develop as well. In the model, there are also transitions from asymptomatic to symptomatic CRC states with the rates depending on the CRC stage [14] (Table 1). We assume in the model that patients receive a colonoscopy once symptoms developed but that the presence of symptoms does not affect the natural history of neoplasia.

#### 2.1.2. Mortality Rate Model

Patient death rates due to both CRC-related mortality and all-cause mortality were considered in the POMP model. Mathematical models for the rates of both processes were developed.

**Figure 3:**
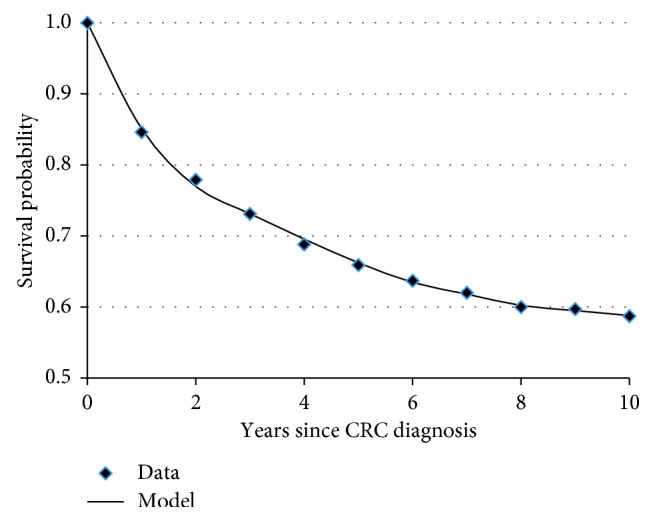
Model fit of overall CRC mortality using breakpoint model.

*(1) CRC-Related Mortality Rates*. The CRC-related mortality rate was simultaneously fit to two SEER data tables [15]: (1) the five-year survival data (2003–2009) for local, regional, and distant CRC for white males based on SEER 18 areas follow-up into 2010 (Table 2) and (2) the annual overall CRC survival rate for 10 years after diagnosis for all patients in 2003 (Figure 3). A least-squares objective function was used to fit every data point from both data sources (4 data points for 5-year survival and 11 data points for overall CRC survival rate).

The CRC-related mortality rate varies by CRC stage so a different mortality rate was used for each CRC stage. The probability of surviving for a period of time *t* after being diagnosed with the *j* ^th^ stage of CRC (where *j* is either local, regional, distant, or unknown), *P*_survivor_^(*j*)^(*t*), is described by

(2) $$\begin{array}{c} \frac{dP_{\text{survivor}}^{\left(j\right)}\left(t\right)}{dt}=-k^{\left(j\right)}\left(t\right)P_{\text{survivor}}^{\left(j\right)}\left(t\right),\\ P_{\text{survivor}}^{\left(j\right)}\left(0\right)=1, \end{array}$$

where *k* ^(*j*)^(*t*) is the mortality rate.

With those probabilities, the overall survival probability of CRC patients with time, *P*_survivor_(*t*), was calculated by combining the survival rates for each stage weighted by the prevalence of each stage:

(3) $$\begin{array}{c} P_{\text{survivor}}\left(t\right)=\frac{0.44P_{\text{survivor}}^{\left(\text{local CRC}\right)}\left(t\right)+0.34P_{\text{survivor}}^{\left(\text{regional CRC}\right)}\left(t\right)+0.18P_{\text{survivor}}^{\left(\text{distant CRC}\right)}\left(t\right)+0.05P_{\text{survivor}}^{\left(\text{unknown CRC}\right)}\left(t\right)}{1.01}. \end{array}$$

A constant mortality rate was not a good fit to the data because there was an overprediction in the earlier years since diagnosis and an underprediction in the latter years. A single breakpoint model was then applied on the mortality rates of each stage of the disease and provided an extremely accurate fit to the data (Figure 3 and Table 3). The mortality rate increased as the CRC spread and the decrease in the mortality rate after a few years matches the expectation that the mortality rate is highest immediately after CRC diagnosis. In addition, the breakpoint was early as the cancer became more distant (7.3, 5.3, and 1.6 years for local, regional, and distant CRC, respectively).

**Figure 4:**
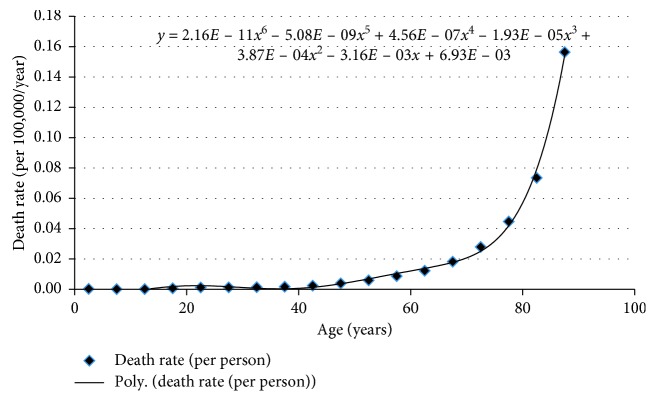
All-cause mortality data.

*(2) All-Cause Mortality*. The all-cause mortality data were obtained from Table 3 in the National Vital Statistics (NVS)—life table database, 2010 [16] (Figure 4). The midpoint of each age range was obtained and plotted against the death rates from the NVS life table database. A sixth-order polynomial was used to fit with the existing National Vital Statistics data. The mortalities for ages above 90 years were extrapolated based on this polynomial.

#### 2.1.3. Partially Observed Markov Process (POMP) Model

Prior to screening colonoscopy, the adenoma growth and mortality rate models are applied to the patients to simulate the likelihood of adenomas and CRC. A patient can die from natural causes, remain in a healthy state, or develop symptoms from CRC. The patients who develop symptoms or die from natural causes are removed from the patient pool because each hypothetical patient is considered healthy until their screening colonoscopy. To mimic the clinical classification of colonoscopy results [12], the probabilities of every possible combination of colonic neoplasia were used to calculate the probability of eight colonic neoplasia states: (1) no adenomas, (2) 1-2 nonadvanced adenomas only, (3) 3+ nonadvanced adenomas only, (4) 1-2 adenomas with some large/advanced adenomas, (5) 3+ adenomas with some large/advanced adenomas, (6) local CRC, (7) regional CRC, and (8) distant CRC.

A partially observed Markov process model was used to obtain the CRC predictions. Because colonoscopy is not 100% sensitive [12], the actual state of the colon is only partially observed during colonoscopy. Multiple studies [5, 12, 13, 17–19] have shown that smaller adenomas are more commonly missed as opposed to larger ones. To be consistent with the adenoma growth model, the sensitivities of Sherer et al. [12] were used; for example, this study reported colonoscopies are more likely to be sensitive for larger adenomas (95.8%) as opposed to diminutive ones (39%).

After a screening colonoscopy, patients will follow a follow-up colonoscopy regimen based on the findings at the screening colonoscopy and patient age. Patients can die from a natural cause at any point in the model and are removed from the pool of patients. The probabilities collected from the POMP model are incorporated into the costs and QALYS results to perform the cost-effectiveness analysis.

### 2.2. Follow-Up Colonoscopy Scenarios

Different follow-up colonoscopy protocols were applied and the associated costs and benefits (in terms of QALYs gained) calculated. We first evaluated one follow-up colonoscopy to determine its effects. Scenarios were evaluated for screening colonoscopy at ages 50, 55, 60, 65, 70, or 75 years, and a single follow-up colonoscopy from 2 years until 20 years in increments of 2 years. The cost per QALY gained was calculated, and the follow-up intervals were identified. The CRC predictions were applied to the follow-up colonoscopy scenarios, and the probabilities collected were incorporated into the costs and QALYS results.

#### 2.2.1. Control Group

For a fair comparison, to measure the effect of follow-up colonoscopy, a control group who received a screening but no follow-up colonoscopy was used. The costs and QALYs were obtained for patients who received surveillance colonoscopies versus those who just had a screening and no follow-up colonoscopy.

### 2.3. Costs

Two types of costs were considered: costs associated with receiving colonoscopy and costs associated with CRC treatment [20–24] (Table 4). We assumed all other costs to be equal between patients.

The cost of screening colonoscopy ranged from $300 to $2,627 [20–24] but most of the colonoscopy costs were around $1,100. An average US price of $1,068 was used. There was also an additional cost of $92.06 due to adverse events [21]. Multiplying the price of each adverse event by its rate and summing over all the adverse events calculated the cost due to adverse events. The types of adverse events incorporated were perforation, serosal burn, bleed with transfusion, bleed without transfusion, and postpolypectomy hemorrhage. The two costs were combined to obtain the overall cost of colonoscopy where *P*_colonoscopy_(*t*, *τ*, **τ**_up_) is the probability of receiving a follow-up colonoscopy at age *t* with screening colonoscopy at age *τ* and follow-ups at ages **τ**_up_, where *P*_colonoscopy_(*t*, *τ*, **τ**_up_)=1 at scheduled colonoscopies and 0 < *P*_colonoscopy_(*t*, *τ*, **τ**_up_) < 1 when colonoscopies are not scheduled due to symptoms. The overall cost of colonoscopy was obtained by

(4) $$\begin{array}{c} {\text{costs}}_{\text{colonoscopy}}\left(t,\tau ,\tau_{\text{up}}\right)=P_{\text{colonoscopy}}\left(t,\tau ,\tau_{\text{up}}\right)\left[\text{cost of colonoscopy}+\text{cost of adverse effects}\right]. \end{array}$$

Initial, surveillance, and terminal [20–24] costs were included within the model for each of the three cancer stages (local, regional, and distant). A weighted average was calculated for each cancer stage based on the different costs obtained [20–24]. CRC is mostly characterized by stages but in our model, we assumed local CRC to be the equivalent of stage 0 and 1 CRC combined, regional CRC to be equivalent to stage 2 and 3 CRC combined, and distant CRC to be equivalent to stage 4 CRC. To further support the numbers we used for the costs, we tested our expected costs against projected costs for CRC for 2020 [25], and they were relatively similar to the 2020 projected costs ($204,445 vs $195,276). To calculate expected costs, we used the weighted averages for the cancer stages multiplied by the prevalence of each cancer stage and divided by the overall sum of the likelihood of each stage of cancer.

All costs were discounted at an annual rate of 3%. The discounting factor at each age was calculated by

(5) $$\begin{array}{c} \text{discounting factor}\left(t,\tau \right)=r\left(\;\;\frac{1}{r}-\frac{1}{re^{r\left(t-\tau \right)}}\;\;\right),\;\; \end{array}$$

where *r* is the discounting rate. The discounting factor was used in the colonoscopy costs to give an updated cost of

(6) $$\begin{array}{c} \text{discounted}\;\;{\text{costs}}_{\text{colonoscopy}}\left(t,\tau ,\tau_{\text{up}}\right)={\text{costs}}_{\text{colonoscopy}}\left(t,\tau ,\tau_{\text{up}}\right)-\left({\text{costs}}_{\text{colonoscopy}}\left(t,\tau ,\tau_{\text{up}}\right)\times \text{discounting factor}\left(t,\tau \right)\right). \end{array}$$

The discounted costs associated with treatment for local, regional, and distant CRC were calculated in a similar manner. If CRC is discovered due to either a scheduled follow-up colonoscopy or colonoscopy due to symptoms, for the first year after CRC diagnosis, all the costs for screening and diagnosis were incorporated and multiplied by the probability of having local cancer at that age. After screening and diagnosis, the patient goes into the first year of surveillance where surveillance costs for local CRC are added to local CRC costs and multiplied by the probability of having local cancer. This calculation continued for the surveillance years with the probability of local cancer changing for each year. For local terminal costs, the calculations were similar to the surveillance, but instead we used the probability of the patient dying with local cancer at each age multiplied by the probability of local CRC. Costs were calculated similarly for regional CRC and distant CRC. Total costs included both the costs of colonoscopy and CRC-related costs:

(7) $$\begin{array}{c} \text{Total costs}\left(\tau ,\tau_{\text{up}}\right)=\overset{100\text{ years}}{\underset{t=0\text{ years}}{\sum }}\left(\text{discounted }\;\;{\text{costs}}_{\text{colonoscopy}}\left(t,\tau ,\tau_{\text{up}}\right)+\text{discounted }\;\;{\text{costs}}_{\text{CRC}}\left(t,\tau ,\tau_{\text{up}}\right)\right), \end{array}$$

where

(8) $$\begin{array}{c} \text{discounted}\;\;{\text{costs}}_{\text{CRC}}\left(t,\tau ,\tau_{\text{up}}\right)=\underset{\text{stage CRC}}{\sum }\left[\text{discounted }{\text{costs}}_{\text{initial,stage CRC}\;\;}\times P_{\text{stage CRC}}\left(t,\tau ,\tau_{\text{up}}\right)+\text{discounted }{\text{costs}}_{\text{surveillance,stage CRC}}\times P_{\text{stage CRC}}\left(t,\tau ,\tau_{\text{up}}\right)+\text{discounted }{\text{costs}}_{\text{terminal,stage CRC}}\times P_{\text{stage CRC}}\left(t,\tau ,\tau_{\text{up}}\right)\times P_{\text{death stage CRC}}\left(t,\tau ,\tau_{\text{up}}\right)\right], \end{array}$$

where the CRC-related costs are summed over the CRC stages of local, regional, and distant.

### 2.4. QALYs

In order to calculate the QALYs, the utilities were obtained from Ness et al. [21] (Table 5). The disease-specific health state utilities were multiplied by the probability of being in that state at each given age. This was calculated by using

(9) $$\begin{array}{c} \text{QALYs}\left(t,\tau ,\tau_{\text{up}}\right)=\underset{\text{state}}{\sum }{\text{QALYs}}_{\text{state}}\times P_{\text{state}}\left(t,\tau ,\tau_{\text{up}}\right), \end{array}$$

which is the weighted average of the QALYs of the potential states of healthy, dead, local CRC, regional, and distant CRC. The QALYs were also discounted at 3% using the same discounting factor in Section 2.3. The discounting factor was used in the QALYs to give updated QALYs of

(10) $$\begin{array}{c} \text{discounted QALYs}\left(t,\tau ,\tau_{\text{up}}\right)=\text{QALYs}\left(t,\tau ,\tau_{\text{up}}\right)-\left(\text{QALYs}\left(t,\tau ,\tau_{\text{up}}\;\;\right)\times \text{ discounting factor}\left(t,\tau \right)\right). \end{array}$$

The discounted QALYs at each age were summed up for all the health states over all ages to obtain the total QALYs following a screening colonoscopy at age *τ* and follow-up(s) at ages **τ**_up_:

(11) $$\begin{array}{c} \text{total QALYs}\left(\tau ,\tau_{\text{up}}\right)=\overset{100\text{ years}}{\underset{t=0\text{ years}}{\sum }}\text{discounted QALYs}\left(t,\tau ,\tau_{\text{up}}\right). \end{array}$$

### 2.5. Cost-Effectiveness Analysis

The POMP model was applied to the follow-up scenarios in Section 2.2 to determine the intercolonoscopy interval that maximizes patient's QALYS per unit cost of colon-related expenses. The primary outcome of a cost-effectiveness analysis is the incremental cost-effectiveness ratio or the cost per QALY gained. This is calculated as the difference in the expected cost of two interventions, divided by the difference in the expected QALYs produced by the two interventions. In order to calculate cost per QALY gained per person, we used

(12) $$\begin{array}{c} \frac{\Delta \text{Cost}}{\Delta \text{QALY}}=\frac{\text{costs of surveillance colonoscopy}-\text{costs of screening colonoscopy only}}{\text{QALYs gained for surveillance colonoscopy}-\text{ QALYs gained for screening colonoscopy only}}. \end{array}$$

The outcome that yielded the greatest increase in QALYs per unit cost of CRC expenses is the optimal solution.

Over the years, there has been an increase in articles referencing both $50,000/QALY and $100,000/QALY as the society's willingness to pay (WTP) threshold for a quality-adjusted life year (QALY) [26, 27] so this fueled our urge to use a $50,000, $75,000, and $100,000/QALY threshold. In the analysis, effectiveness was the change in QALYs, and the efficiency referred to the change in cost per QALY.

### 2.6. Sensitivity Analysis

We performed a one-way sensitivity analysis to test the effect changes in various fundamental assumptions (the cost of colonoscopy, the cost of cancer, and the discount rate) would have on the cost-effectiveness. The analysis involves changing one key factor and then repeating the exact steps done previously. The ranges used in the sensitivity analysis were based on literature review [20–24] (Table 4). For the sensitivity analysis on the discount rate, we tested a 0% discount rate.

## 3. Results

### 3.1. Cost-Effectiveness of Follow-Up Colonoscopy by Screening Age and Screening Colonoscopy Results

Follow-up colonoscopy was only cost-effective after screening colonoscopy at age 50 years and at the $100,000/QALY gained threshold. Follow-up colonoscopy for screening that occurs after the age of 50 years was not cost-effective, and no scenario was cost-effective at the $75,000/QALY gained or the $50,000/QALY gained thresholds.

**Figure 5:**
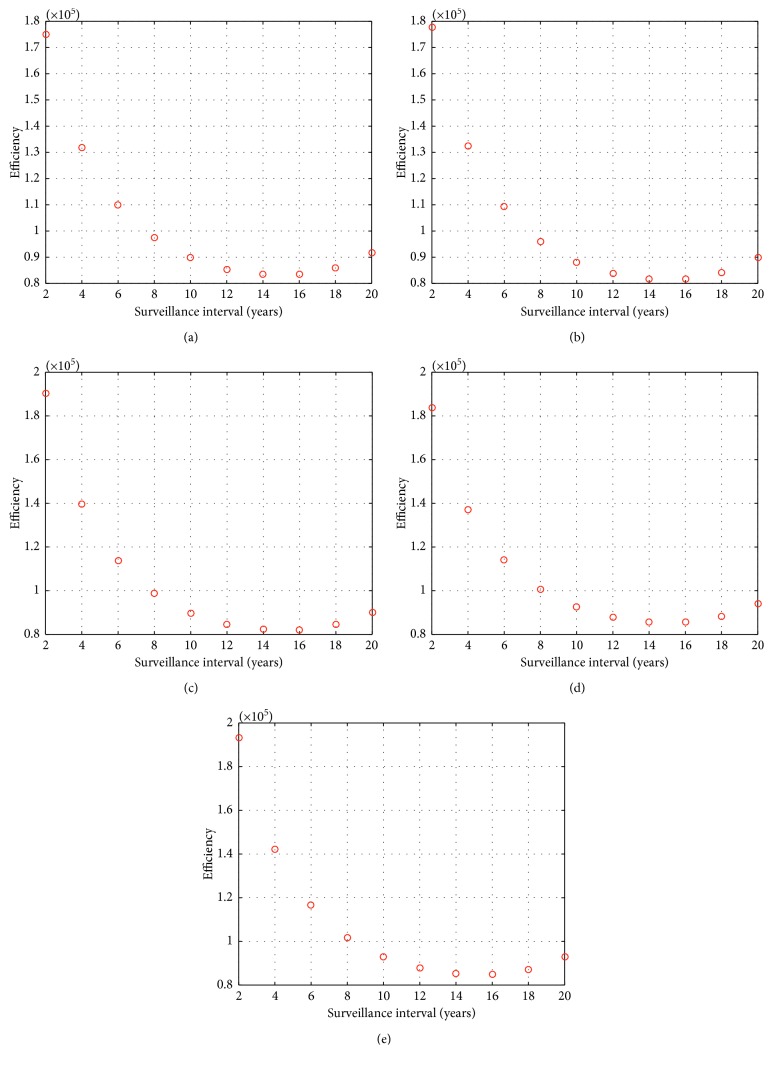
Efficiency vs surveillance intervals at age 50 for screening colonoscopy findings of (a) no neoplasia, (b) 1 to 2 polyps only, (c) 3 plus polyps only, (d) 1 to 2 advanced polyps only, and (e) 3 plus advanced polyps only.

At age 50, one follow-up colonoscopy was cost-effective. In Figure 5, all the follow-up colonoscopies (2 to 20 years later) at age 50 for all screening findings can be seen. Only the $100,000 per QALY threshold is cost-effective. At screening age 50, no cost-effective follow-up colonoscopies were recorded for the other two thresholds, and there is nothing represented below $80,000.

### 3.2. Cost-Effective Intercolonoscopy Intervals That Maximize QALYs Gained by Age and Screening Results

**Figure 6:**
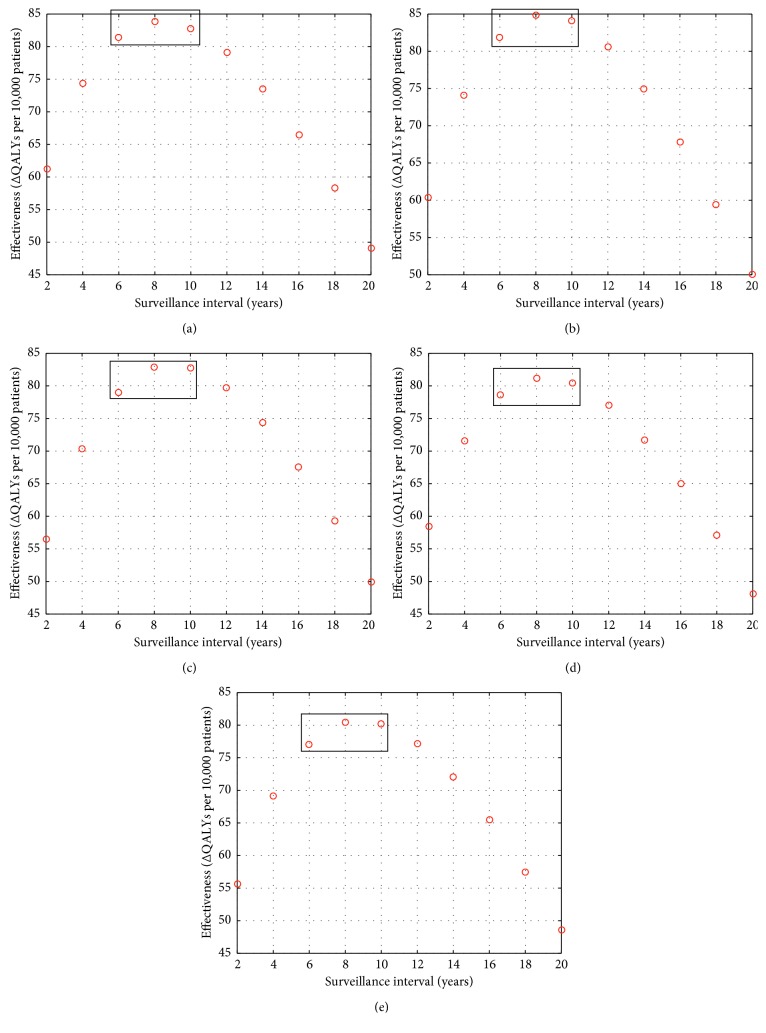
Effectiveness vs surveillance intervals (ΔQALY) at age 50 with (a) no neoplasia, (b) 1 to 2 small polyps only, (c) 3 plus small polyps only, (d) 1 to 2 advanced polyps only, and (e) 3 plus advanced polyps only. The three points that were used for the interpolation are highlighted in the boxes. The maximum falls somewhere between 6 and 10 years.

From the pool of follow-up intervals that were cost-effective, the one that produced the most QALYs was considered optimal. A quadratic interpolation was done—using the maximum point and the two points surrounding the maximum—to determine the interval that yielded the most QALYS (see Figure 6, for an example).

The intercolonoscopy intervals were largely insensitive to the screening colonoscopy results: intercolonoscopy intervals stratified by the screening result were all within a 0.5-year range (Table 6). Following screening at age 50 years, the most QALYs gained at the $100,000/QALY threshold was one follow-up colonoscopy with an average interval of 8.5 years. This follow-up resulted in 84.0 QALYs gained per 10,000 patients.

### 3.3. Sensitivity Analyses

The effects of changing the values of key factors in the model were relatively insensitive to high screening colonoscopy costs and low cancer cost but sensitive to lower screening colonoscopy costs, higher cancer cost, and the 0% discount rate (Table 7).

When the higher end of the colonoscopy cost was used, nothing was cost-effective at any screening age at any of the three thresholds. When the low end of the colonoscopy cost was used, more screening ages (55, 60, and 65 years) were cost-effective at the $100,000/QALY gained threshold, and the interval for screening age 50 years extended from 7.7 to 20 years to 2 to 20 years. With the screening ages that were cost-effective, the recommended follow-up intervals and number of QALYS gained/10,000 persons decreased with increasing age.

When using the low end of the cost of cancer, at screening age 50 years, the cost-effective window was recorded between 10 and 20 years at the $100,000/QALY threshold, compared to 7.7 to 20 years from the original analysis. Also the recommended follow-up interval dropped from 8.5 years to 8.3 years, and there was a 0.1 decrease in QALYS gained/10,000 persons. Nothing became cost-effective at the $75,000/QALY gains or $50,000/QALY gained thresholds. With the high end of cancer cost, follow-up colonoscopy after screening at age 55 years became cost-effective 10 to 14 years later with an average 5.8 years follow-up interval and 75.6 QALYS gained/10,000 persons. Also with the high end of cancer cost, the cost-effective interval for follow-up colonoscopy after screening at age 50 years was not very significant compared to the original analysis (7.7 to 20 years vs 8 to 20 years). The same follow-up interval and QALYs gained were observed as from the original analysis.

With the 0% discount rate, it showed that as many as four follow-up colonoscopies were cost-effective at screening age 50 at the $100,000/QALY threshold with 437.2 QALYs gained/10,000 persons. More screening ages also became cost-effective at the $50,000 and $75,000/QALY thresholds (Table 7).

## 4. Discussion

The optimal intercolonoscopy interval at the $100,000/QALY gained threshold following a screening colonoscopy at age 50 years of approximately 8.5 years is in the neighborhood of the current guideline of a 10-year intercolonoscopy interval for an average risk patient [3]. However, the guideline also applies to screening colonoscopies after the age of 50 years while we found that follow-up colonoscopy for screening at ages 55 years and older is not cost-effective. This result is influenced by the selection of the discounting rate. The 3% discount rate accounts for the perceived value of money (and is the standard when performing cost-effectiveness analyses), but a 0% discount is the true monetary cost-effectiveness.

With a 0% discount rate, we saw as many as four follow-up colonoscopies being cost-effective at the $100,000/QALY threshold, which is more consistent with clinical recommendations. After the four follow-up colonoscopies, patients were 68.8 years, which is consistent with the USPTF recommendation that screening ends at 75 years [6] and clinical studies showing that the benefits of colonoscopies decrease with age [6, 28]. The average life expectancy in the US is approximately 79 years, so the impact of CRC prevention and early detection due to colonoscopy will likely be minimal. In addition, after 75 years, patients tend to have more health issues, and undergoing colonoscopy may not be the most comfortable experience plus the rewards would not be significant as discussed earlier. From the sensitivity analysis, we also saw that with lower screening colonoscopy costs and higher cancer costs, we get follow-up colonoscopies at later ages.

We found that the 8.5-year intercolonoscopy interval is consistent across screening colonoscopy results while clinical recommendation becomes shorter for more advanced findings [29]. The shortening of clinical recommendations is based on data that advanced neoplasia are more prevalent following screening colonoscopy with advanced neoplasia [10, 29, 30]. The mathematical model also predicts a higher risk of CRC in patients with advanced neoplasia but, in the simulations, most of the patients who develop cancer receive follow-up colonoscopy due to symptoms prior to the recommended interval. Because of the inclusion of colonoscopy due to symptoms, the systematic follow-up of all patients at an earlier time is not necessarily cost-effective on the population level.

Not surprisingly, the number of follow-up colonoscopies will increase if one is willing to pay more for a unit of health. From increased amounts in what one is willing to spend, we can see that more colonoscopies can occur leading to shorter intercolonoscopy intervals. For decades, $50,000/QALY gained has been used as a benchmark for the cost of a unit of health [26, 27, 31]. Using the benchmark of $50,000/QALY gained may be somewhat outdated due to inflation and economic growth [27]. It is a reasonable enough cost but based on our study was not cost-effective. Based on the sensitivity analysis, we can see that with increasing threshold on the societal willingness to pay for a QALY, more follow-up colonoscopies are possible with higher QALYs gained/10,000 persons. By spending more, you can receive more benefit. It should be noted that some patients would spend more than $100,000/QALY gained to ensure better health. The threshold used is solely based on patient's values and how they approach risk.

More benefit would mean more QALYs gained from more colonoscopies. The more the tests one can get, the more the information that can be gathered to aid in preventing or reducing CRC incidence [4, 32]. Cost and benefit are directly proportional as shown in the study. More patients are undergoing screening colonoscopy and will have a lifetime of potential follow-up colonoscopy. Early follow-up colonoscopy in patients who are at high risk is extremely crucial [33]. Recommendations for follow-up after screening colonoscopy are an important indicator of a patient's future health. Keeping track of patient's health status through surveillance can detect any abnormalities early on and treat it as needed.

A limitation of the study is that the results rely on an adenoma growth model developed using data from the Veterans Affairs (VA) medical system so, while this model is accurate for this population, the population contained few female and nonwhite patients. Outside of the VA, the results obtained may not be applied in a broad aspect but the methodology and model basis can be applied to datasets with similar characteristics. Currently, there is no other dataset with adenoma growth rates that could be used for validation, but it will be important to validate the results with a broader population.

An important feature of this study is the evaluation of the cost-effectiveness of the individual follow-up colonoscopies rather than a screening protocol as a whole. Previous studies have evaluated the cost-effectiveness of a single colonoscopy [21] or an entire protocol [22, 24]. A single screening colonoscopy between 50 and 54 years of age is very cost-effective with Ness et al. calculating its cost-effectiveness at less than $10,000/additional QALY gained, for example. The screening is so cost-effective that additional costs for additional treatments can be absorbed—even if there is minimal in QALYs—and the protocol will remain cost-effective. Both a screening colonoscopy and follow-up colonoscopy creates a gain in QALY but, due to the CRC preventive ability of colonoscopy, the gain in QALY is less for each follow-up colonoscopy. The current study calculates the marginal cost-effectiveness of each follow-up colonoscopy to determine whether each additional follow-up colonoscopy is cost-effective rather than the entire protocol.

## 5. Conclusions

The current study is the first to quantitatively analyze the frequency of follow-up colonoscopies based on age and findings from screening results. Our results indicate that patients should follow-up colonoscopies sooner than the 10 years recommended by the USPTF. We obtained results quantifying those intervals based on age at screening and also based on the findings at that screening. With advancing technology and medical improvements, spending more for a unit of life if you can is not a bad idea. We can safely say, continuing screening after the age 75 years is not recommended, as the benefit at that point is little to nothing. Follow-up colonoscopies may give more life years, given the appropriate protocol based on age and findings from screening.

## Figures and Tables

**Table 1:** Annual transition probabilities between adenoma and CRC states.

	Yearly transition probability
*Adenoma growth transition*	
No adenoma to diminutive	Varies from 0 to 0.0971 depending on age
Diminutive to no adenoma	0.0181
Diminutive to medium adenoma	0.0163
Medium to diminutive adenoma	0.0716
Medium to large adenoma	0.0377
Medium adenoma to CRC	0.0025
Large to medium adenoma	0.0047
Large adenoma to local CRC	0.0028

*CRC growth transition*	
Local CRC to regional CRC	0.22
Regional CRC to distant CRC	0.50

*CRC symptom transition*	
Asymptomatic local CRC to symptomatic local CRC	0.17
Asymptomatic regional CRC to symptomatic regional CRC	0.22
Asymptomatic distant CRC to symptomatic distant CRC	0.50

**Table 2:** Prevalence and 5-year survival rates of CRC by stage at diagnosis for patients diagnosed in the US [15].

CRC stage	Prevalence (%)	CDC data for 5-year survival rate (%)	Model-predicted 5-year survival rate (%)
Local	44	88.2	88.1
Regional	34	70.1	70.0
Distant	18	12.3	12.2
Unknown	5	43.1	43.1

**Table 3:** Initial and secondary mortality rates applied to the model for each stage of the disease.

CRC stage	Initial mortality rate (1/year)	Breakpoint (years)	Second mortality rate (1/year)
Local	0.025	7.3	0.018
Regional	0.068	5.3	0.025
Distant	0.600	1.6	0.085
Unknown	0.155	4.8	2.2*E* − 06

**Table 4:** Costs used in the cost-effectiveness analysis.

Type of treatment	Costs (range for sensitivity analysis)
*Costs of colonoscopy*	
Colonoscopy	$1,068.59 ($303–$2627) [20–24]
Adverse effects	$92.06 [21]
*Annual cost of CRC treatment*	
Local	
Initial (year 1)	$20,247.20 ($13,848–$25,527.02) [20–24]
Surveillance (years 2–5)	$1305.04 ($425–$2353.26) [20–24]
Regional	
Initial (year 1)	$26,007.50 ($15,398–$37639.27) [20–24]
Surveillance (years 2–5)	$2346.72 ($1424–$4014.69) [20–24]
Distant	
Initial (year 1)	$30085.20 ($17,223–$42,401) [20–24]
Surveillance (years 2–5)	$15057 ($2702–$26,855) [20–24]
Terminal (if CRC results in mortality)	$23,002.35 ($11,188–$50,920) [20–24]

**Table 5:** QALYs used in the cost-effective analysis.

Patient status	Annual QALY utility
Healthy	0.91
Dead	0
Local CRC	0.74
Regional CRC	0.70
Distant CRC	0.25

**Table 6:** Recommended follow-up colonoscopy intervals by colonoscopy screening age and screening colonoscopy results.

Screening colonoscopy age and results	Recommended follow-up interval	Range of cost-effective follow-up colonoscopy	Gain in QALYs/10,000 persons for recommended follow-up interval
$100,000/QALYs gained threshold			
50 years			
All findings	8.5 years	7.7–20 years	84.0
No neoplasia	8.5 years	7.6–20 years	84.0
1 to 2 nonadvanced adenomas only	8.5 years	7.4–20 years	84.0
3+ nonadvanced adenomas only	9.0 years	7.8–20 years	83.3
1 to 2 adenomas with some advanced neoplasia	8.4 years	8.2–20 years	81.3
3+ adenomas with some advanced neoplasia	8.9 years	8.4–20 years	80.7

**Table 7:** Intercolonoscopy intervals for sensitivity analyses.

Screen age	Outcome	Colonoscopy cost		Cancer cost		Discount rate
		Low ($303)	High ($2,627)	Low	High	
$100,000/QALYs gained						

50 years	Recommended follow-up interval	8.5 years	n/c^*∗*^	8.3	8.5	3.9, 4.0, 4.9, 6 years (437.22)
	Range of cost-effective follow-up colonoscopy	2–20 years		10–20 years	8–20 years	
	Gain in QALYs/10,000 persons	84.0		83.9	84.0	
55 years	Recommended follow-up interval	6.8	n/c^*∗*^	n/c^*∗*^	5.8	3.6, 4.5, 5.8 years (305.52)
	Range of cost-effective follow-up colonoscopy	2–20 years			10–14 years	
	Gain in QALYs/10,000 persons	75.1			75.6	
60 years	Recommended follow-up interval	5.6 years	n/c^*∗*^	n/c^*∗*^	n/c^*∗*^	6.4 years (120.01)
	Range of cost-effective follow-up colonoscopy	2–20 years				
	Gain in QALYs/10,000 persons	62.6				
65 years	Recommended follow-up interval	4.3 years	n/c^*∗*^	n/c^*∗*^	n/c^*∗*^	n/a
	Range of cost-effective follow-up colonoscopy	2–14 years				
	Gain in QALYs/10,000 persons	47.6				

$75,000/QALYs gained						
50 years		n/c^*∗*^	n/c^*∗*^	n/c^*∗*^	n/c^*∗*^	6.4, 8.1 years (307.30)
55 years		n/c^*∗*^	n/c^*∗*^	n/c^*∗*^	n/c^*∗*^	8.2 years (157.14)

$50,000/QALYs gained						
50 years		n/c^*∗*^	n/c^*∗*^	n/c^*∗*^	n/c^*∗*^	10.3 years (194.12)

^*∗*^n/c scenarios that were not cost-effective

## Data Availability

The cancer patient data used to support the findings of this study are available from the corresponding author upon request.
